# 

**Published:** 1898-12-03

**Authors:** 


**Notices of Births, Deaths, and Marriages** are inserted in this column at a charge of 2s. 6d. for an announcement not exceeding four lines.

### NOTICE TO CORRESPONDENTS.

All MS., Letters, Books for Review, and other matters intended for the Editor should be addressed The Editor, "The Hospital," The Hospital Building, 28 & 29, Southampton Street, Strand, W.C.

The Editor cannot undertake to return rejected MS., even when accompanied by stamped directed envelope.

**Notice to Advertisers and Subscribers.**

All Advertisements, Orders for Copies of The Hospital, and Business Communications should be addressed to **THE MANAGER (Not to the Editor),** The Hospital, The Hospital Building, 28 & 29, Southampton Street, Strand, London, W.C.

The Cost of Subscription, post free, is as follows:—Per week, 2½d.; per quarter, 2s. 8d.; per half-year, 5s. 3d.; per annum, 10s. 6d. Foreign:—Per annum, 15s. 2d., payable, in all cases, in advance.

**NOTICE.—Vol. XXIV. of "The Hospital" (April, 1898, to September, 1898), in handsome cloth binding, is now on sale at the Office of this Journal, price 7s. 6d. Cases for binding the Numbers contained in this Volume 1s. 6d. Index to Vol. XXIV., 2½d., post free.**

**The Monthly Part for December is now ready. Price 6d., by post 8d.**

### BOOKS RECEIVED.

Shaw and Sons.

"Shaw's Manual of the Vaccination Law." By a Barrister-at-Law. Sixth edition.

C. A. Pearson (Limited).

"Mad Humanity: Its Forms Apparent and Obscure." By L. Forbes Winslow, D.C.L. Oxon, M.B., LL.M. Camb.

James Bowden.

"An Idyll of the Dawn." By Mr. Fred Reynolds.

Sampson Low, Marston, and Co.

"A New Astronomy." By David P. Todd, M.A., Ph.D.

### Scraps and Gleanings.

Hospital Sunday in Devizes has this year shown an encouraging revival, over £85 having been placed to the credit of the hospital funds, as against £20 odd from the same source last year.

The Lord Mayor of Birmingham has suggested that the whole of the Birmingham charities should benefit each year by the Birmingham Hospital Sunday Fund, and he is endeavouring to get his idea put into practice by the authorities.

The question of the site for the Bexhill Cottage Hospital is still undecided. The committee have again met, suggested a different site from the one under consideration at the previous meeting, and, after some fruitless discussion, have again adjourned the meeting. Still another site will apparently have now to be selected.

### The Student of Medicine.

### APPOINTMENTS.

G. H. Ahrens, appointed House Surgeon of the Royal Ear Hospital. W. O. Beddard, M.R.C.S.Eng., L.R.C.P.Lond., appointed House Surgeon to the Dover Hospital. A. N. Brushfield, M.R.C.S., L.R.C.P., appointed Senior House Surgeon to the Macclesfield Infirmary. E. T. Burton, M.R.C.S.Eng., L.R.C.P.-Lond., appointed Resident Medical Officer at the Workhouse Infirmary, Birmingham. F. A. H. Clarke, M.R.C.S.Eng., L.R.C.P.Lond., appointed Assistant Medical Officer at the Beech Avenue Workhouse, Nottingham. A. W. German, M.R.C.S., L.R.C.P.Lond., appointed Medical Officer for the North Municipal District of the West Derby Union. A. H. Gibbon, L.R.C.P., L.R.C.S.Edin., appointed Medical Officer for the St. Mary's District of the Bury St. Edmunds Incorporation. W. T. Grant, M.B., C.M.Edin., appointed Delegate for Sweden and Norway to the Quarantine Board of Egypt. H. H. Heffernan, M.R.C.S.-Eng., L.R.C.P.Lond., appointed Medical Officer for the Woolston District of the Rugby Union. T. N. Kelynack, M.D., M.R.C.P., appointed Honorary Pathologist and Bacteriologist to the Manchester Ear Hospital. J. R. McKinlay, M.R.C.S., L.R.C.P.-Lond., L.S.A., appointed Assistant Medical Officer to the Gordon Road Workhouse and the Heaton Road Homes of the Parish of St. Giles's, Camberwell. J. H. Morgan, M.A.Oxon., F.R.C.S., appointed Examiner in Surgery at the University of Oxford. A. H. Pritchard, M.R.C.S.Eng., L.R.C.P., appointed House Surgeon to the Cancer Hospital, Brompton, S.W. O. F. Rowley, M.R.C.S.Eng., L.R.C.P.Lond., appointed Honorary Surgeon to the Beckett Hospital and Dispensary, Barnsley. C. R. Scott, M.B.Edin., appointed Assistant Medical Officer to the Warneford Asylum, Oxford. A. R. Sieveking, L.R.C.P., L.R.C.S.Edin., L.F.P.S.Glasg., appointed Senior Medical Officer to the Uganda Railway, East Africa. T. W. Stanton, M.R.C.S.Eng., L.R.C.P.-Lond., appointed Medical Officer for the Osburnby District of the Sleaford Union. G. C. Walker, jun., M.B., Ch.B.Vic., M.R.C.S.Eng, appointed Resident Medical Officer and House Physician to the Royal Mineral Water Hospital, Bath. R. P. Williams, M.B.Lond., appointed House Surgeon to the Hospital for Sick Children, Great Ormond Street.

### PASS LIST.

### University of London.

M.B. EXAMINATION, OCTOBER, 1898.

First Division.—C. W. Buckley, St. Mary's Hospital; H. Minnie Castledine, B.Sc., Royal Free Hospital; F. F. Elwes, Middlesex Hospital; J. G. Emanuel, B.Sc., Mason College and Queen's and General Hospitals, Birmingham; H. Fitz-Neville Hine, Middlesex Hospital; T. J. Horder, St. Bartholomew's Hospital; H. Innes, London Hospital; H. G. Lawrence, St. Mary's Hospital; F. S. Lloyd, St. Mary's Hospital; D. N. Nabarro, B.Sc., University College; W. B. H. Wood, Queen's and General Hospitals, Birmingham.

Second Division.—W. M. Anderson, London Hospital; J. Ashton, St. Mary's Hospital; H. T. Barron, Westminster Hospital; S. H. Belfrage, St. Thomas's Hospital and University College; J. W. H. Bendle, St. Mary's Hospital; T. P. Berry, Guy's Hospital; G. P. Bletchly, Middlesex Hospital; Elizabeth H. Bone, Royal Free Hospital; Gabrielle R. S. Breeze, London School of Medicine and Royal Free Hospital; F. Brickwell, St. Bartholomew's Hospital; F. Butterfield, Owen's College and Manchester Royal Infirmary; V. E. Collins, Guy's Hospital; Mabel E. Cousins, Royal Free Hospital; H. A. T. Fairbank, Charing Cross Hospital; Lucinda C. E. Forster, Royal Free Hospital; G. D. Freer, Queen's and Mason's Colleges and St. Bartholomew's Hospital; A. B. Fry, London Hospital; L. Gilbert, St. Thomas's Hospital; J. A. Glover, Guy's Hospital; A. S. Green, Royal College of Surgeons, Ireland, and Meath Hospital; W. L. Griffiths, B.Sc., University College; J. Grimshaw, London Hospital; S. Gross, Yorkshire College and General Infirmary, Leeds; J. P. Hall, Owen's College and Manchester Royal Infirmary; L. E. C. Handson, Guy's Hospital; H. E. Hewitt, St. Thomas's Hospital; J. Howell, Guy's Hospital; R. C. Leaning, St. Mary's Hospital; F. C. Lewis, St. Mary's Hospital; C. D. Lindsey, St. Mary's Hospital; J. P. Maxwell, St. Bartholomew's Hospital; P. W. Moore, Guy's Hospital; D. J. Munro, Guy's Hospital; H. Peet, Yorkshire College; A. G. G. Plumley, Medical School and University College, Bristol, and Guy's Hospital; C. S. Read, University College; F. Riley, Westminster Hospital; Adeline Mary Roberts, London School of Medicine and Royal Free Hospital; P. W. Rowland, St. Bartholomew's Hospital; C. Runcle, St. Mary's Hospital; H. A. Schölberg, St. Bartholomew's Hospital; H. D. Singer, St. Thomas's Hospital; C. A. Sprawson, King's College; R. H. J. Swan, Guy's Hospital; G. P. Tayler, St. Bartholomew's Hospital; W. H. M. Telling, Guy's Hospital; C. J. Utley, Owen's College; A. Young, University College, Sheffield and London.

JUST OUT.

Crown 8vo., Paper Covers, 1s.,

SOME HEALTH ASPECTS OF EDUCATION.

By PERCY G. LEWIS, M.D., M.R.C.S., L.S.A.,

Author of "Nursing: Its Theory and Practice."

London: The Scientific Press, Ltd., 28 & 29, Southampton Street, Strand, London, W.C.

